# U.S. Montmorency Tart Cherry Juice Decreases Bone Resorption in Women Aged 65–80 Years

**DOI:** 10.3390/nu13020544

**Published:** 2021-02-07

**Authors:** Tiffany Dodier, Kendall L. Anderson, James Bothwell, Janice Hermann, Edralin A. Lucas, Brenda J. Smith

**Affiliations:** Department of Nutritional Sciences, Oklahoma State University, Stillwater, OK 74078, USA; tiffany.dodier@okstate.edu (T.D.); kendall.anderson@okstate.edu (K.L.A.); james.bothwell.jr@gmail.com (J.B.); janice.hermann@okstate.edu (J.H.); lucas.a.lucas@okstate.edu (E.A.L.)

**Keywords:** tart cherry, osteoporosis, bone mass, bone resorption, oxidative stress, inflammation, aging

## Abstract

Pre-clinical studies have demonstrated that tart cherries, rich in hydroxycinnamic acids and anthocyanins, protect against age-related and inflammation-induced bone loss. This study examined how daily consumption of Montmorency tart cherry juice (TC) alters biomarkers of bone metabolism in older women. Healthy women, aged 65–80 years (*n* = 27), were randomly assigned to consume ~240 mL (8 fl. oz.) of juice once (TC1X) or twice (TC2X) per day for 90 d. Dual-energy x-ray absorptiometry (DXA) scans were performed to determine bone density at baseline, and pre- and post-treatment serum biomarkers of bone formation and resorption, vitamin D, inflammation, and oxidative stress were assessed. Irrespective of osteoporosis risk, the bone resorption marker, tartrate resistant acid phosphatase type 5b, was significantly reduced with the TC2X dose compared to baseline, but not with the TC1X dose. In terms of indicators of bone formation and turnover, neither serum bone-specific alkaline phosphatase nor osteocalcin were altered. No changes in thiobarbituric acid reactive substances or high sensitivity C-reactive protein were observed in response to either TC1X or TC2X. We conclude that short-term supplementation with the higher dose of tart cherry juice decreased bone resorption from baseline without altering bone formation and turnover biomarkers in this cohort.

## 1. Introduction

Osteoporosis is a widespread public health concern that afflicts more than 200 million people worldwide [1,2]. Alarmingly, women account for 61% of the 9 million osteoporosis-related fractures that occur annually [1]. The first 5–10 years post menopause, a stage of unstable decreases in the bone-preserving hormone estrogen, is characterized by a rapid phase of bone loss and is a major contributor to the high prevalence of osteoporosis amongst women [3]. This phase of rapid bone loss is followed by a more gradual decline in bone mass in the decades that follow as declining estrogen stabilizes and the rate of bone turnover slows. Activity of the cells that determine the rate of bone turnover, (i.e., osteoclasts, osteoblasts, and osteocytes) is largely driven by cytokines and mediators of oxidative stress that are altered in response to this decline in estrogen [4]. Although there are effective FDA-approved pharmacological interventions available to treat osteoporosis, there is a need for alternative strategies that minimize concerns with side-effects associated with existing agents (e.g., osteonecrosis of the jaw and atypical femoral fractures), which can negatively impact patient compliance [5,6]. Plant-based foods, long recognized for their health and medicinal properties, could offer a novel dietary approach to prevent bone loss associated with aging by targeting inflammatory and oxidative processes [6]. Fruits, which provide a rich source of hydroxycinnamic acids and anthocyanins (e.g., certain varieties of blueberries and dried plums), have been studied for their capacity to reduce oxidative stress and inflammation, and stand out for their osteoprotective activity [7,8,9,10].

Tart cherry (*Prunus cerasus*) is another fruit that is particularly high in these osteoprotective polyphenols. We reported [11] that supplementing the diet with lyophilized tart cherry (5 and 10% *w*/*w* diet) increased whole-body bone mineral density (BMD) and improved trabecular and cortical microarchitectural parameters in a mouse model of age-related bone loss. Similar improvements in microarchitectural parameters and bone strength were observed in a transgenic tumor necrosis factor (TNF)-α model of rheumatoid arthritis with the same doses of tart cherry supplementation [12]. In these reports, the mechanisms through which changes in bone structural parameters occurred were attributed to increased bone mineralization and decreased bone resorption. This anti-resorptive property of tart cherry is supported by in vitro studies demonstrating that tart cherry polyphenols decrease the number of tartrate-resistant acid phosphatase (TRAP)-positive multinucleated cells and TRAP activity, consistent with a decrease in both the differentiation and activity of osteoclasts [13].

Tart cherry has also been investigated for its health benefits in clinical trials associated with cardiovascular disease, muscle recovery, sleep quality, and osteoarthritis [14,15,16,17,18]. In addition to these reported health benefits, tart cherry juice has been shown to decrease biomarkers of inflammation, such as high sensitivity C-reactive protein (hsCRP), as well as indicators of oxidative stress such as thiobarbituric acid reactive species (TBARS) [15,19,20,21,22,23]. The pronounced effect of tart cherry supplementation observed in age-related bone loss coupled with the promising results of clinical trials to reduce inflammation and oxidative stress motivated our interest in determining if the osteoprotective properties translate to humans.

This study aimed to determine the effect of tart cherry juice supplementation, at two different doses, on biochemical markers of bone formation and resorption in women aged 65–80 years. Due to reports that tart cherry juice has potent anti-inflammatory and antioxidant activity, and the role that these biological processes play in bone loss, we also investigated the effects of two different doses of tart cherry juice on serum indicators of oxidative stress and inflammation. We hypothesized that 90 days of daily tart cherry juice supplementation would decrease biomarkers of bone resorption compared to baseline in a dose-dependent manner and that the response would be greater in women with higher risk for osteoporosis. Furthermore, these alterations in biomarkers of bone metabolism would coincide with a decrease in markers of oxidative stress and inflammation.

## 2. Materials and Methods

### 2.1. Participants

**Figure 1:**
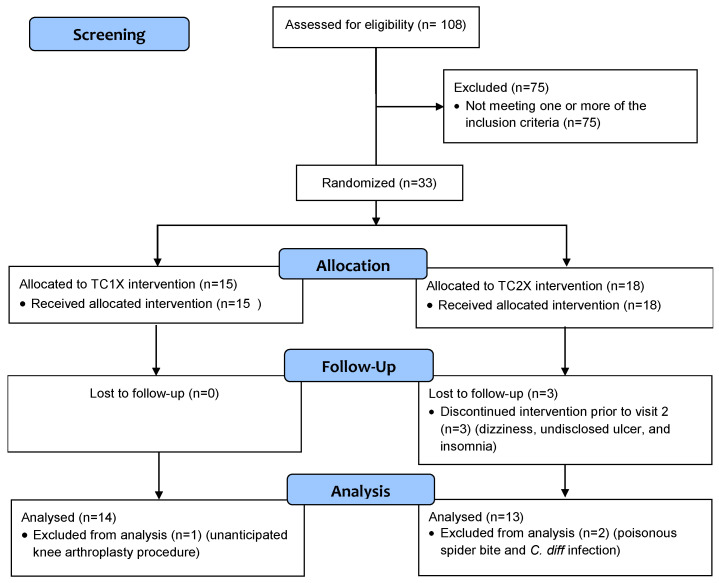
Study Flow Diagram.

Volunteers were recruited through email flyer distribution to university-associated and community group email lists, as well as posting at university and area community wellness programs, senior centers, and local clinics. Women (*n* = 108) were screened and thirty-three women aged 65–80 years qualified to participate in the study based on the inclusion and exclusion criteria (Figure 1). In addition to age, inclusion criteria consisted of women able to ambulate without assistance and have the capacity to give informed consent. Exclusion criteria included current smokers, individuals with a body mass index (BMI) <18.5 or >40 kg/m^2^ based on self-reported height and weight, and individuals with a previous diagnosis of osteoporosis or any other metabolic bone disease, renal disease, cancer, cardiovascular disease, diabetes mellitus, pulmonary disease, gastrointestinal diseases, liver disease, or other chronic conditions that could affect bone metabolism. Additionally, subjects were excluded if they had taken hormone replacement therapy or other medications or supplements known to alter bone or calcium metabolism (e.g., bisphosphonates, denosumab, raloxifene, intermittent parathyroid hormone, growth hormone, steroids, natural estrogens) within three months of starting treatment. All procedures were approved by the Oklahoma State University Institutional Review Board in accordance with the ethical standards of the institutional and with the 1964 Helsinki Declaration and its later amendments or comparable ethical standards. This study was registered with ClinicalTrials.gov: NCT04167150.

### 2.2. Study Design

Participants who qualified for the study visited the Nutritional Sciences Clinical Research Laboratory three times (i.e., baseline, 45 days, and 90 days). The 90-day study duration was based on previous studies using tart cherry to determine its anti-inflammatory and antioxidant properties, and the investigation of other dietary interventions with fruits designed to study bone biomarkers [9,15]. At the baseline visit, participants reviewed and signed the informed consent with a member of the research team, completed a fasted blood draw, underwent anthropometric measures, dual-energy x-ray absorptiometry (DXA) scans (i.e., hip, spine and whole body), hand grip strength testing, and completed a series of questionnaires collecting information about their medical history, physical activity, sun exposure, and calcium intake. Participants were randomized to one of two treatment arms (TC1X = 240 mL tart cherry juice once per day; TC2X = 240 mL tart cherry juice twice per day) at their initial baseline visit. The tart cherry juice concentrate, produced from U.S.-grown Montmorency tart cherries, provided 70 kcal per serving (30 mL), with 19 g of carbohydrate (15 g total sugar), 15 mg of sodium, and ~225 gallic acid equivalents of total phenolics. The doses of tart cherry juice used in this study had been shown to have anti-inflammatory and antioxidant effects [15]. Each participant sampled the juice at the baseline visit to confirm no adverse allergic reactions and were given a 45-day supply of the study product. Instructions were provided on how to reconstitute the concentrate into a juice (i.e., 30 mL concentrate to ~210 mL water), with disposable measuring cups provided and informed that they could choose to consume the concentrate as a syrup. Participants were instructed to consume the test product at the same time each day with approximately eight hours between doses for the TC2X group (i.e., morning and evening, at least three hours before sleep to prevent reflux). Participants were also instructed to record the time(s) they consumed the product on a calendar that was provided to assess compliance. At the second visit, participants reported any changes in their health status or medications, height and weight were recorded, compliance calendar returned, and the remainder of the test product was provided. The third or final visit was similar to the initial visit: measuring anthropometrics, completing questionnaires, returning a three-day food record and having a fasted blood draw. The DXA scan provided context as to the current osteoporosis risk of participants, but was not repeated at the final visit, given that the 90-day study duration was not expected to yield discernable changes in bone density.

### 2.3. Medical History, Physical Activity, Calcium Intake and Sun Exposure Questionnaires

Health and medical history information was collected at baseline by research personnel using a medical history questionnaire. Changes in medical status were reported at the second and final visits. Information provided included basic demographics (e.g., age, ethnicity, education), lifestyle factors (e.g., tobacco and alcohol use), as well as other current and historical information pertinent to bone health (e.g., prior use of hormone replacement therapy (HRT), pregnancies and breastfeeding, menopause onset, fractures, surgeries, and previous medical diagnoses). Also, included in the questionnaire was a list of prescription and over-the-counter medications and supplements taken in the past three months with respective dosages.

Physical activity was assessed using the Yale Physical Activity Survey (YPAS) [24]. The YPAS is specifically designed for administration to older adults and assesses recent (i.e., previous seven days) physical activity and physical activity patterns over the previous month. The survey provides estimated activity and total calorie expenditure for the previous week, as well as monthly index scores for total activity of the previous month and categories (e.g., vigorous activity and leisurely walking). Energy expenditures were used to categorize participant activity. Light activity was characterized as participation in daily exercise that expends ~150 kcal in excess of those expended during activities of daily living, whereas active individuals expend ~250 kcal/day in excess. The monthly index scores are a product of weighted values of frequency and duration for given activities. At the baseline visit, study participants were asked to maintain their current level of physical activity over the 90-day study period.

Dietary intake of macronutrients and vitamin D was assessed using three-day food records. Participants were asked to maintain their regular dietary intake throughout the study and were instructed on how to record food intake by research personnel using portion size references which were completed after their initial visit and prior to their final visits. Usual supplemental and dietary intake of calcium was assessed at the baseline and final visits using the National Institutes of Health (NIH) Short Calcium Questionnaire (version SCQ 2002) [25]. The three-day food records were analyzed using Food Processor II (ESHA Research, Salem, OR, USA) to assess dietary intake.

Sun exposure was determined using a validated questionnaire [26] that assigns a sun exposure score (0–56) of the previous week based on duration in the sun or a tanning bed and amount of unprotected skin. Furthermore, participants classified their skin type from six descriptions to qualify the sun exposure effect.

### 2.4. Anthropometric Measurements

Height, weight, and waist and hip circumferences were measured at the initial and final visits using the protocol outlined by the NHANES III from which body mass index (BMI, kg/m^2^) and waist-to-hip ratio were calculated [27]. Total body soft tissue analysis using a DXA system (QDR 4500 Discovery A, Hologic, Waltham, MA, USA) provided body composition, total fat-free mass less mineralized bone, and fat mass at baseline to support hand-grip strength and physical activity assessments, as well as to qualify BMD measures.

DXA scans were performed at the baseline visit by a certified bone densitometrist. Specific measurements included hip (total, neck, trochanter, and intertrochanter) and lumbar spine (L1-L4) bone mineral density (BMD), bone mineral content (BMC), and bone mineral area (BMA). T- and Z-scores were determined using Hologic software. Assessment of BMD and T-scores provided important insight into the participants’ osteoporosis risk at baseline.

As a functional indicator, muscle strength was assessed at the baseline and final visits with a hand grip dynamometer (Jamar, Lafayette, IN, USA) using a previously published protocol [28]. Briefly, the participant was seated with their feet flat on the floor, arms on the armrests of a chair, and the weight of the dynamometer supported by a member of the research staff. Participants performed the hand grip test three times with both their dominant and non-dominant hand, exerting maximal effort each time. The best of three hand grip strength tests for both dominant and non-dominant hand was used as the indicator for muscle strength and function.

### 2.5. Serum Biomarkers

Fasting blood samples were collected by staff at the OSU University Health Services Clinic during baseline and final visits. Blood samples were collected in vacutainer tubes, allowed to coagulate at room temperature and then centrifuged at 4000× *g* for 20 min. Serum was separated and aliquoted for storage at −80 °C.

Biomarkers of bone formation, resorption and turnover were assessed on serum samples collected at baseline and final visits. Bone alkaline phosphatase (BAP), an indicator of bone formation, was assessed using EIA kits from Immunodiagnostic Systems Inc. (IDS, Gaithersberg, MD, USA). Serum total osteocalcin (i.e., N-terminal-mid-fragment and intact OCN) and tartrate-resistant acid phosphatase type 5b (TRAcP 5b) were also assessed using ELISA kits from IDS as indicators of bone turnover and bone resorption, respectively. All assays were run in duplicate and strictly adhered to the manufacturer’s protocol.

Serum biomarkers of inflammation, oxidative stress, and vitamin D status were also determined at baseline and final visits. hsCRP, a biomarker of inflammation, was measured using a commercially available kit (Carolina Liquid Chemistries Corp, Winston Salem, NC, USA) on a Biolis 24i Clinical Chemistry Analyzer. Serum TBARS, an indicator of oxidative stress, was assessed using a Cayman Chemical kit (Ann Arbor, MI, USA). Vitamin D status (i.e., 25-hydroxy vitamin D [25-OH-VitD]) was determined using an IDS EIA kit.

### 2.6. Statistical Analysis

All data were analyzed using PC SAS version 9.4 (SAS Institute, Cary, NC, USA). Descriptive statistics (e.g., means, standard errors, medians, minima, and maxima) were calculated for continuous variables. Normality of continuous variables was assessed using Shapiro–Wilk’s test for normality. The difference between baseline and final, the primary outcome of serum bone biomarkers, and secondary outcome that included indicators of oxidative stress and inflammation, was assessed using paired *t*-test. Next, differences between baseline and final serum bone biomarkers were assessed after adjusting for serum 25-OH vitamin D and osteoporosis risk, which included baseline whole body, hip, femur neck, and spine BMD or T-score at the hip, femur neck, and spine as covariates. Categorical data for baseline characteristics, T-scores, BMI, and vitamin D status were assessed using Chi square analyses followed by confirmation with Fisher’s exact test to determine if differences existed between the two groups. For all analyses, alpha was set to 0.05 and data are presented as means ± standard error (SE) unless otherwise specified.

## 3. Results

### 3.1. Participant Demographic and Lifestyle Factors

Thirty-three participants were enrolled in the study, but three withdrew before the second visit due to complaints of dizziness, exacerbation of an undisclosed ulcer, and interference with sleep (Figure 1). Of the 30 participants that completed their study visits, three participants were excluded from the analysis for reasons unrelated to the study (i.e., one participant excluded due to an unanticipated knee arthroplasty procedure, and two participants excluded due to the need to undergo extensive treatments for a poisonous spider bite and serious gastrointestinal *C. difficile* infection). Of the 27 participants remaining, compliance was >90% and did not differ between groups. The study population was primarily Caucasian (96%), married (70%), and highly educated (44% reported post-graduate education) with a mean age of 70.9 ± 4.5 years (Table 1). Self-reported mean years post-menopause was 23.0 ± 5.6 years for the study population overall with no difference between the TC1X and TC2X groups. The two treatment groups were similar in age and distribution of ethnicity, marital status, and education (Table 1).

With regard to lifestyle factors, 74% of participants reported that they consume alcohol, but the mean number of drinks per week was 1.8 ± 2.4, which is well below the recommended limit for women (i.e., no more than one drink/day) (Table 1) [29]. At baseline, there were no differences between treatment groups for the proportion of participants who consumed alcohol, the number of drinks consumed, and those who reported ever smoking. Reproductive history, including gravidity, parity, and years of oral contraceptive use, were similar between treatment groups at baseline (Table 1).

Baseline BMD T-scores obtained from DXA scans revealed that 82% of the women were osteopenic at one or more sites (Table 2). The frequency of osteopenia and osteoporosis in the femur neck for the study population was *n* = 20 and *n* = 3, respectively. The distribution of osteoporosis categories between TC1X and TC2X treatment groups tended to be different at the femur neck (*p* = 0.088), but was similar between groups at all other sites. There were no participants in the TC2X group who had a normal femur neck T-score and 50% more with osteopenia compared to TC1X. No significant differences were observed in the distribution of T-scores considered normal (T-score > −1.0), osteopenia (T-score −1 to −2.5), or osteoporosis (T-score < −2.5) between TC1X and TC2X groups at the hip, hip sub-regions, or lumbar spine. Specific bone parameters (i.e., BMD, BMC, and BMA) were not different between groups at baseline (Supplementary Materials Table S1).

### 3.2. Anthropometric, Diet Analysis and Physical Activity

In characterizing baseline anthropometrics of participants, the overall study population distribution of BMI by category was 44% (*n* = 12) normal, 37% (*n* = 10) overweight, and 19% (*n* = 5) obese, with no differences in distributions between the TC1X and TC2X groups (Table 3). There were no differences in weight, height, BMI, percent body fat, waist circumferences, and waist-to-hip ratio between treatment groups at baseline or final time points. At the final visit, weight and BMI were significantly greater than baseline within the TC1X group (1.3% increase, *p* = 0.0203 and 1.4% increase, *p* = 0.0158, respectively). However, participants in the TC1X group were still within the same category from baseline (i.e., overweight) and waist circumference was not different from baseline (Table 3). There were no statistically significant differences in any anthropometric measures within the higher dose (i.e., TCX2) group or between groups.

To determine if changes in dietary intake occurred during the course of the study, the average macronutrient and vitamin D intakes were calculated from three-day food records and calcium intake was determined from the calcium questionnaire. The mean intakes of total kcal and percent of kcal from carbohydrates, fat, and protein were not different at baseline or final time points between TC1X and TC2X treatment groups (Table 3). In general, both groups consumed 15–20% of their calories from protein, 40–50% from carbohydrates, and 30–40% from fat at baseline and final visits. The percentage change in total kcal intake and contribution of protein, carbohydrates, and fats in the diet were not significantly different within and between TC1X and TC2X treatment groups. The tart cherry juice provided an additional 70 kcal and 19 g of carbohydrates per day to the TC2X group compared to the TC1X group [30]. Calcium intake was examined at baseline and final visits using the calcium questionnaire. In this cohort, total calcium intake, as well as intake from supplements or food, was not different between TC1X and TC2X treatment groups at either time point and the proportion of participants not meeting the recommended dietary allowance (RDA) for calcium was similar between groups (Table 3).

Dietary intake of vitamin D from food and from dietary supplements was similar between groups at baseline and final time points and within each treatment group (Table 3). Due to the potential influence of physical activity and muscular strength on bone homeostasis, energy expenditure and physical activity were estimated and hand grip strength was assessed at the baseline and final visits. Based on self-report, the study cohort was categorized as lightly active with a mean daily exercise EE of more than 200 kcal/day (i.e., 1468 ± 262 kcal/week) (Table S2). Neither exercise nor total energy expenditure (EE) differed between TC1X and TC2X treatment groups at the baseline or final time points. Within treatment groups, total EE tended to be lower at the final visit for the TC2X group (*p* = 0.0575), as did self-reported vigorous (*p* = 0.010) and leisure walking (*p* = 0.002) activity scores. The summary moving index score was not different between the TC1X and TC2X treatment groups; but there was a decrease in the summary index score within the TC2X group (Table S2). Overall, the values for hand grip strength obtained from this cohort were within the normal range for women in this age group [28]. Grip strength was similar between groups at the baseline visit and was not altered over the course of the study when comparisons were made within and between treatment groups (Table S2).

### 3.3. Vitamin D Status

In addition to the dietary intake of vitamin D, sun exposure was assessed using a validated questionnaire at both the baseline and final visits. No differences in sun exposure score were revealed between groups at baseline (TC1X = 18.4 ± 2.9 vs. TC2X = 21.1 ± 3.3) and final time points. However, at the final time point, the TC2X mean sun score was significantly lower than the baseline visit score (TC2X Baseline = 21.1 ± 3.3 vs. Final = 13.4 ± 3.3; *p* = 0.0455), but were not different between groups (TC1X = 17.6 ± 2.4 vs. TC2X = 13.4 ± 3.3). A score > 14 indicates that the participant spent > 30 min outside with at least hands and face exposed daily over the previous week.

There were no differences in serum 25-OH VitD between the TC1X and TC2X groups at baseline, nor were there differences within or between groups after the 90-day treatment period (Table 4). According to the Institute of Medicine (IOM), Food and Nutrition Board guidelines [31], nine out of the 27 participants in this cohort were considered at risk for vitamin D deficiency; however, the distribution of these participants was not different between groups at either time point (Table 4).

### 3.4. Serum Bone Formation and Resorption Indicators

**Figure 2:**
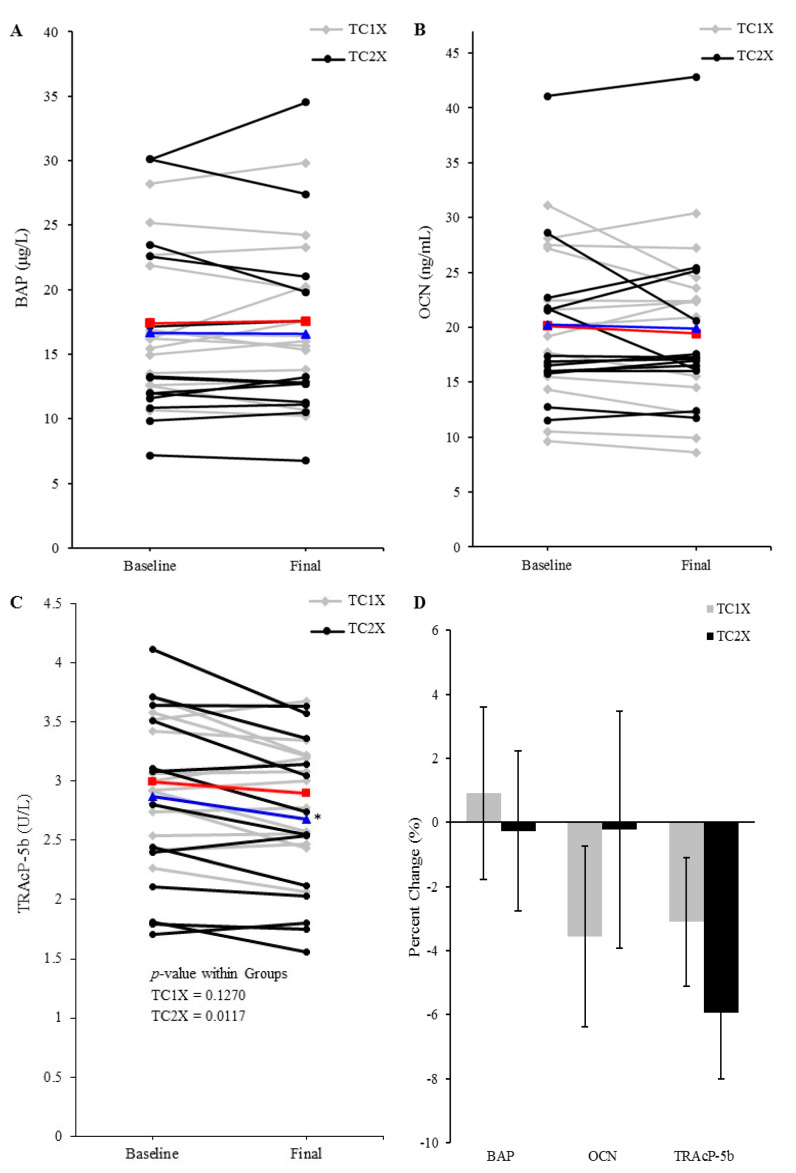
Effect of 90-day tart cherry juice treatment on serum bone biomarkers: (**A**) bone-specific alkaline phosphatase (BAP), (**B**) osteocalcin (OCN), and (**C**) tartrate-resistant acid phosphatase (TRAcP)-5b. Baseline and final values are shown for individual participants and the group mean is presented as TC1X = red line or TC2X = blue line. Serum BAP and OCN were unchanged within groups for both the TC1X and TC2X, but serum TRAcP-5b was decreased (*p* < 0.05) from baseline to final visit (*) within the TC2X group, but not the TC1X group. (**D**) Percent change from baseline was not different between TC1X and TC2X groups for any of the biomarkers of bone metabolism that were assessed.

Analysis of serum samples from baseline and final visits provided insight into tart cherry juice’s effects on bone formation, turnover, and resorption. Serum BAP, an indicator of bone formation, was not significantly different between groups at the baseline (TC1X = 17.40 ± 5.15 U/L vs. TC2X = 16.41 ± 7.67 U/L) or at the final (TC1X = 17.58 ± 5.48 U/L vs. TC2X = 16.27 ± 7.72 U/L) visit. Likewise, there was no difference in serum OCN, considered a biomarker of bone turnover, between treatment groups at baseline (TC1X = 20.11 ± 6.64 μg/L vs. TC2X = 19.88 ± 7.83 μg/L) or final (TC1X = 19.41 ± 6.65 μg/L vs. TC2X = 19.67 ± 8.04 μg/L) time points. The effect of tart cherry within treatment groups yielded no significant alterations in either serum BAP or OCN over the course of this 90-day study (Figure 2A,B). Serum TRAcP 5b, an indicator of resorption, was not significantly different between groups at baseline (TC1X = 2.99 ± 0.46 U/L vs. TC2X = 2.79 ± 0.81 U/L) and final (TC1X = 2.89 ± 0.45 U/L vs. TC2X = 2.60 + 0.71 U/L) visits. Although there was no effect of TC1X on bone resorption, TRAcP 5b was reduced (*p* = 0.0117) by TC2X after 90 days (Figure 2C). Percent change from baseline was not different between TC1X and TC2X groups for any of the biomarkers of bone metabolism that were assessed (Figure 2D). In addition to assessing the effects of TC1X and TC2X within treatment group over time using paired *t*-test, we also investigated whether adjusting for osteoporosis risk or vitamin D status (25-OH VitD) would alter our results. Irrespective of a participant’s baseline BMD and T-score at the spine, hip, and hip subregions, or vitamin D status, no alteration in BAP and OCN (*p* > 0.05) occurred in response to tart cherry juice. Furthermore, a participant’s osteoporosis risk and vitamin D status at baseline did not influence the decrease in TRAP5b (*p* < 0.05) with the TC2X dose.

### 3.5. Serum Indicators of Inflammation and Oxidative Stress

General determinants of inflammation and oxidative stress were assessed based on serum hsCRP and TBARS, respectively. Baseline hsCRP for the cohort (2.2 ± 0.5 mg/L) was within the normal reference range (<3 mg/L) and similar between the TC1X and TC2X treatment groups at both the baseline and final visits (Table 4). No alterations were observed in hsCRP within either treatment group. Serum TBARS were similar between groups at baseline and final time points (Table 4), but the mean values were slightly higher in this cohort than the normal reference range (1.86–3.94 μM) [32]. No significant changes in serum TBARS were noted within either the TC1X or TC2X groups.

## 4. Discussion

Identifying promising dietary interventions that can be incorporated into a lifestyle program to reduce the risk for osteoporosis is an appealing strategy. This study was designed to investigate whether chronic consumption of the juice of tart cherries, a fruit with osteoprotective effects in pre-clinical studies and no known side effects in human studies, alters serum biomarkers of bone formation and resorption in postmenopausal women, 65–80 years of age. As anticipated, this cohort of women with a mean age of 71 years was in large part osteopenic (82%); 11% of the population was osteoporotic at one or more sites, and only 7%, or two participants, were considered to have normal bone density (T-score > −1) at all sites assessed. Two doses of tart cherry juice were utilized in this study that had been shown to alter indicators of oxidative stress and inflammation in previous studies [15,33,34].

In this cohort, there were no alterations in biomarkers of bone formation, bone turnover or bone resorption in response to the lower dose of tart cherry juice (TC1X), but the higher dose (TC2X) significantly reduced the resorption biomarker, TRAcP 5b, over the 90-day study period. This reduction in bone resorption occurred without any alterations in serum indicators of bone formation and turnover, which can be a drawback to pharmaceutical agents with anti-resorptive activity such as bisphosphonates [35]. Furthermore, it occurred despite the decline in physical activity, both vigorous and leisurely walking activity, reported by this group. Because this was a pilot study to determine if tart cherry juice altered bone metabolism, and if so the dose that would be required, the study was not designed with a duration that would be required to observe a change in BMD. However, we anticipated that osteoporosis risk (i.e., BMD or T-scores) and vitamin D status of the study participants at baseline may affect their response to treatment. Our results showed that when controlling for these factors, there were no changes in the findings related to the biomarkers, suggesting that tart cherry juice at the higher dose reduced bone resorption in this cohort of older postmenopausal women, irrespective of their bone density and 25-OH vitamin D status. Similar decreases in TRAcP 5b have been reported in clinical trials with dried plum, which is important due to the fruit being in the same genus and having a similar phytochemical profile as tart cherry [9,36,37,38].

In previous studies utilizing animal models of osteoporosis, lyophilized Montmorency tart cherry powder from cherries grown in the same region of the U.S. as those used to produce the juice in this study, was incorporated into the diet. The tart cherry increased BMD in an animal model of age-related bone loss, which resulted from improved trabecular bone microarchitecture in the lumbar spine and distal femur metaphysis, and enhanced cortical thickness in the mid-diaphysis of the femur [11]. No systemic biochemical markers of bone formation or resorption were observed in that study, but alterations in genes involved in bone mineralization (i.e., phosphate regulating endopeptidase homolog, X-linked or *Phex* and peroxisome proliferator activated receptor or *Ppar-γ*) were reported with tart cherry treatment. In previous work by Moon and colleagues using a TNF-α overexpressing transgenic model [38], tart cherry supplementation improved trabecular and cortical bone parameters. Although systemic indicators of bone metabolism were not evaluated in the study, local RANKL and TRAP expression, indicators of osteoclast differentiation and activity, were significantly reduced in the transgenic mice with tart cherry treatment. Runt-related transcription factor 2 (*Runx2*) gene expression was also increased in transgenic TNF mice by tart cherry supplementation, but no alterations in collagen type I expression. These findings suggest that tart cherry promoted the differentiation of osteoblasts without altering their activity.

Because no previous clinical studies had investigated the effects of the lyophilized tart cherry powder on bone-related outcomes, and only tart cherry juice has been shown to alter indicators of inflammation and oxidative stress [15,39,40], the juice was utilized in this study. The loss of bioactive components of the tart cherry, such as the polyphenolic compounds, is minimal during heat treatment used in preparing juice concentrates [41]. However, other bioactive components within the fruit, such as non-digestible carbohydrates, that could contribute to the bone protective effects of the tart cherry due to their influence on microbiota-derived short chain fatty acids and mineral absorption, may not be preserved in the juice preparation to the same level as the powder [41,42]. Thus, the use of the juice may account for the discrepancies in the skeletal response in this study compared to our previous work with animal models of age-related bone loss.

Tart cherry juice has been shown to have potent antioxidant and anti-inflammatory properties in studies focused on athletes, otherwise healthy adults with osteoarthritis, obese and overweight individuals, as well as older adults [18,22,23,33,34]. Since oxidative stress and pro-inflammatory mediators promote osteoclast activity resulting in an increase in bone resorption, we also investigated whether tart cherry juice alters indicators of oxidative status and inflammation in our cohort. Although TRAcP 5b was decreased by the higher dose of tart cherry juice in this study compared to baseline, there were no differences in serum hsCRP or TBARS, systemic indicators of inflammation and oxidative stress, respectively. A general expectation is that inflammation and oxidative stress increase with age, but some studies reporting improvements in indicators of inflammation and oxidative stress with tart cherry have included younger and otherwise healthy participants who were experiencing acute increases in these biomarkers, such as those associated with running a marathon, competing in a triathlon, or high intensity interval training [21,43,44]. Tart cherry juice supplementation may have more pronounced effects in acute scenarios. Studies examining pharmacokinetics of tart cherry phytochemicals reveal that peak concentration of phenolic acid degradation products in plasma occurs 1–2 h post-consumption of ~240–480 mL of tart cherry juice and return to baseline levels within 6-8 h [45,46]. It is not without reason to consider that routine dosing of tart cherry juice, as with twice per day in the TC2X group, may transiently down-regulate inflammation and oxidative stress to reduce osteoclast activity without significantly affecting the markers examined in this study. Alternatively, a recent study that included both men and women, aged 65–80 years, consuming tart cherry juice or control drink (~480 mL per day) for 12 weeks exhibited lower serum hsCRP and malondialdehyde (MDA), one of the end-products of polyunsaturated fatty acid peroxidation, than the control group [34]. However, the upward drift in the control group CRP between baseline and final measurements may have contributed to this observation. In a study of obese and overweight adults, CRP tended to be decreased with 4 weeks of tart cherry supplementation, but the mean participant values were elevated at baseline in that study [47]. In our cohort, the mean CRP was within the normal range at baseline. Population differences could account for these discrepancies in CRP, as well as the specific biomarker of oxidative stress that was used. It should be noted that TBARS include measures of MDA within the sample and the MDA generated from hydroperoxides, which can be affected by triglycerides [48]. However, it is also possible that tart cherry juice influences osteoclast activity through other mechanisms, as has been observed in in vitro studies with tart cherry extracts [13,49].

Despite the interesting finding that tart cherry juice suppresses bone resorption, this study is not without limitations. First, previous pre-clinical studies focused on tart cherry and bone utilized a lyophilized tart cherry powder where this study used tart cherry juice. The decision to use tart cherry juice in this clinical trial was based on its previously shown effects on biomarkers of inflammation and oxidative stress, as well as ease of administration and access. It is possible that the juice preparation has a reduced concentration of bioactive components per serving that are responsible for the osteoprotective effects of tart cherry reported in the animal studies [11,12]. Second, we elected to utilize a pretest—posttest study design with two different doses of treatment as opposed to a placebo-controlled study design with a single dose. We appreciate the limitations of this approach, but in the absence of studies focused on tart cherry and bone in humans and our inability to run three groups that were adequately powered due to budgetary constraints, we decided on the pretest—posttest study design. Future studies with the higher dose of tart cherry juice in a placebo-controlled trial should be performed. Third, with bone biomarkers as the primary outcomes of this study, our power calculation was based on previous reports in the literature with dried plum, which has a similar polyphenolic profile as the tart cherry [45,50]. Our study may have been underpowered to detect small effects on indicators of inflammation and oxidative stress or more subtle changes at lower doses.

In conclusion, this study showed that supplementation with tart cherry juice reduced bone resorption in a cohort of older postmenopausal women. This occurred when the cherry juice was consumed twice per day without changes in biomarkers of bone formation or turnover. These effects on biomarkers of bone metabolism occurred irrespective of the participant’s osteoporosis risk and vitamin D status at baseline. It is clear, that in pre-clinical as well as clinical studies, that tart cherry positively influences bone metabolism in age-related bone loss, but the evidence supporting the mechanism differed. The use of different tart cherry preparations should be considered when comparing the effects of tart cherry on bone metabolism to that of the pre-clinical trial. Thus, the findings of this study can be used to inform the planning of future long-term clinical studies examining the effects of tart cherry juice, but highlight the need to consider the investigation of the skeletal response to the whole fruit.

## Figures and Tables

**Table 1:** Baseline Characteristics of Study Participants.

Characteristic	Total (*n* = 27)	TC1X ^1^ (*n* = 14)	TC2X ^1^ (*n* = 13)	*p*-Value ^2^
Age (years)	70.93 ± 4.47	70.57 ± 4.15	71.31 ± 4.94	0.678
Time Post menopause (years)	22.96 ± 5.58	23.86 ± 8.09	22.00 ± 4.56	0.474
Marital status				0.432
Single (%)	0.07 (2)	0 (0)	15.38 (2)	
Married (%)	70.40 (19)	71.40 (10)	69.20 (9)	
Widowed (%)	14.80 (4)	21.40 (3)	7.70 (1)	
Divorced (%)	14.80 (4)	7.10 (1)	23.10 (3)	
Education				0.409
High school diploma (%)	7.40 (2)	0 (0)	13.33 (2)	
Some college (%)	18.52 (5)	14.29 (2)	23.08 (3)	
College degree (%)	29.63 (8)	23.08 (4)	33.33 (4)	
Postgraduate (%)	44.44 (12)	57.14 (8)	30.77 (4)	
Ethnicity				1.000
Black or African American (%)	3.70 (1)	7.1 (1)	0 (0)	
White (%)	96.30 (26)	92.90 (13)	100.00 (13)	
Lifestyle				
Ever smoked (%)	29.63 (8)	30.77 (4)	28.57 (4)	1.000
Consumes any alcohol (%)	74.07 (20)	71.40 (10)	76.90 (10)	1.000
Frequency (drinks/week)	1.78 ± 2.44	2.34 ± 2.97	1.18 ± 1.62	0.224
Reproductive history				
Gravidity	2.6 ± 0.3	2.9 ± 0.4	2.3 ± 0.4	0.980
Parity	2.1 ± 0.2	2.2 ± 0.3	2.0 ± 0.3	0.562
Oral contraceptive use (years)	8.1 ± 1.9	10.4 ± 3.2	5.7 ± 2.0	0.224

Data are presented as mean ± standard error (SE) unless specified otherwise. ^1^ TC1X = 1 fl. oz. tart cherry juice concentrate/d; TC2X = 2 fl. oz. tart cherry juice concentrate/d. ^2^ *p*-values represent comparison paired *t*-test for continuous data and Chi Square confirmed by Fisher’s Exact test for categorical data between TC1X and TC2X.

**Table 2:** Categorization of Osteoporosis Risk by T-scores Based on dual x-ray absorptiometry (DXA) Scan at Baseline.

Characteristic	Total (*n* = 27)	TC1X ^1^ (*n* = 14)	TC2X ^1^ (*n* = 13)	*p*-Value ^2^
Any site T-score ^3^				0.490
Normal (%)	7.4 (2)	14.3 (3)	0 (0)	
Osteopenia (%)	81.5 (22)	71.4 (10)	92.3 (12)	
Osteoporosis (%)	11.1 (3)	14.3 (2)	7.7 (1)	
Total hip T-score				0.3180
Normal (%)	33.3 (9)	42.9 (6)	23.1 (3)	
Osteopenia (%)	63.0 (17)	50.0 (7)	76.9 (10)	
Osteoporosis (%)	3.7 (1)	7.1 (1)	0 (0)	
Femur neck T-score				0.088
Normal (%)	14.8 (4)	28.6 (4)	0 (0)	
Osteopenia (%)	74.1 (20)	57.1 (8)	92.3 (12)	
Osteoporosis (%)	11.1 (3)	14.3 (2)	7.7 (1)	
Trochanter T-score				1.000
Normal (%)	37.0 (10)	35.7 (5)	38.5 (5)	
Osteopenia (%)	59.3 (16)	57.2 (8)	61.5 (8)	
Osteoporosis (%)	3.7 (1)	7.1 (1)	0 (0)	
Intertrochanter T-score				0.334
Normal (%)	55.6 (16)	64.3 (9)	46.2 (6)	
Osteopenia (%)	40.7 (10)	28.6 (1)	53.8 (7)	
Osteoporosis (%)	3.7 (1)	7.1 (1)	0 (0)	
Lumbar spine T-score				0.265
Normal (%)	51.9 (14)	57.1 (8)	46.2 (6)	
Osteopenia (%)	40.7 (11)	28.6 (4)	53.8 (7)	
Osteoporosis (%)	7.4 (2)	14.3 (2)	0.0 (0)	

^1^ Data are presented as % of participants (*n*) within each category. ^1^ TC1X = 1 fl. oz. tart cherry juice concentrate/d; TC2X = 2 fl. oz. tart cherry juice concentrate/d. ^2^ *p*-values represent Fisher’s Exact test and Chi Square between TC1X and TC2X. ^3^ Considers the lowest T-score at any site and classifies into normal, osteopenia, or osteoporosis T-score ranges.

**Table 3:** Comparisons of Anthropometric Measurements and Macronutrient, Vitamin D and Calcium Intake Within and Between Groups.

Parameter	TC1X ^1^ (*n* = 14)			TC2X ^1^ (*n* = 13)			*p*-Value ^3^	
	Baseline	Final	*p*-Value ^2^	Baseline	Final	*p*-Value ^2^	Baseline	Final
Anthropometrics								
Weight (kg)	66.7 ± 4.1	67.6 ± 4.3	0.020	74.3 ± 3.5	75.4 ± 3.4	0.175	0.176	0.175
Height (cm)	161.8 ± 1.8	161.8 ± 1.7	0.855	163.9 ± 2.3	463.6 ± 2.2	0.331	0.480	0.521
BMI (kg/m^2^)	25.4 ± 1.4	25.7 ± 1.4	0.016	27.5 ± 0.9	28.0 ± 0.8	0.129	0.204	0.177
Normal (%)	8 (57.1)	7 (50.0)	1.00	4 (30.8)	3 (23.1)	1.000	0.522	0.372
Overwt (%)	4 (28.6)	5 (35.7)	-	6 (46.2)	6 (46.2)	-	-	-
Obese (%)	2 (14.3)	2 (14.3)	-	3 (23.1)	4 (30.8)	-	-	-
Waist circ (cm)	84.9 ± 3.3	84.5 ± 3.4	0.598	88.7 ± 2.2	89.8 ± 2.2	0.264	0.364	0.203
Waist/Hip ratio	0.82 ± 0.01	0.81 ± 0.02	0.134	0.83 ± 0.02	0.84 ± 0.02	0.790	0.670	0.296
Body fat (%)	36.3 ± 1.6	-	-	40.1 ± 1.1	-	-	0.163	-
Macronutrients								
TEnergy (kcal)	1729.1 ± 116.8	1931.2 ± 119.3	0.226	1641.8 ± 96.6	1787.9 ± 136.2	0.344	0.573	0.435
Protein (%kcal)	18.0 ± 1.4	15.7 ± 1.0	0.178	17.7 ± 1.6	17.2 ± 1.2	0.757	0.880	0.313
Carbs (%kcal)	46.2 ± 3.1	43.9 ± 2.2	0.390	50.1 ± 2.1	48.6 ± 2.0	0.585	0.313	0.127
TFat (%kcal)	36.9 ± 3.0	40.6 ± 2.2	0.144	34.1 ± 1.4	35.8 ± 1.6	0.392	0.425	0.103
Daily Calcium Intake								
Diet (mg)	942.8 ± 71.2	888.1 ± 84.7	0.425	1091.2 ± 121.9	1079.7 ± 125.6	0.919	0.295	0.212
Supplement (mg)	557.3 ± 110.0	563.9 ± 107.0	0.953	616.7 ± 176.0	523.8 ± 129.7	0.405	0.774	0.812
Total Ca (mg)	1500.1 ± 141.6	1452.1 ± 130.0	0.696	1707.9 ± 191.1	1603.5 ± 199.6	0.502	0.386	0.525
%Below RDA	4 (28.6)	4 (28.6)	1.000	4 (23.1)	6 (46.2)	0.411	1.000	0.440
Daily Vitamin D Intake								
Diet (IU/day)	141.9 ± 25.2	100.7 ± 26.0	0.123	143.2 ± 22.8	115.6 ± 18.1	0.288	0.970	0.646
Supplement (IU/day)	1585.7 ± 515.1	1585.7 ± 515.1	1.000	1076.9 ± 338.0	1076.9 ± 338.0	1.000	0.424	0.424
Total (IU)	1727.6 ± 509.3	1686.4 ± 510.1	0.123	1220.1 ± 345.3	1192.5 ± 341.1	0.288	0.424	0.138

Data are presented as mean ± SE. ^1^ TC1X = 1 fl. oz. tart cherry juice concentrate/d; TC2X = 2 fl. oz. tart cherry juice concentrate/d. ^2^ *p*-values are comparison within groups (baseline vs. final). ^3^ *p*-values represent comparison of TC1X and TC2X groups at baseline or final time points. Body mass index (BMI), Overwt (Overweight), circ (circumference), TEnergy (Total Energy), TFat (Total Fat).

**Table 4:** Effect of 90-day Tart Cherry Juice Consumption on Biomarkers of Vitamin D Status, Inflammation and Oxidative Stress.

Parameter	TC1X ^1^ (*n* = 14)			TC2X ^1^ (*n* = 13)			*p*-Value ^3^	
	Baseline	Final	*p*-Value ^2^	Baseline	Final	*p*-Value ^2^	Baseline	Final
Vitamin D Status								
25(OH)D (ng/mL)	36.7 ± 3.1	35.6 ± 3.2	0.595	31.2 ± 2.3	30.8 ± 2.4	0.783	0.173	0.250
At risk (%) ^4^	4 (28.6)	5 (35.7)	1.000	4 (30.8)	6 (46.2)	0.688	1.000	0.704
Inflammation								
hsCRP (mg/L)	2.2 ± 0.5	2.4 ± 0.6	0.557	2.5 ± 0.7	4.4 ± 0.2	0.593	0.739	0.642
Oxidative Stress								
TBARS (μM)	4.6 ± 0.5	3.9 ± 0.5	0.180	5.1 ± 0.6	2.8 ± 0.6	0.235	0.545	0.388

Data are presented as mean ± standard error (SE). ^1^ TC1X = 1 fl. oz. tart cherry juice concentrate/d; TC2X = 2 fl. oz. tart cherry juice concentrate/d. ^2^ *p*-values represent comparison within groups between baseline and corresponding final values. ^3^ *p*-values represent comparison between TC1X and TC2X at baseline and final values. ^4^ At risk for vitamin D deficiency = 12–30 ng/mL; no participants were deficient (i.e., 25(OH)D < 12 ng/mL). High sensitivity C-reactive protein (hsCRP), thiobarbituric acid reactive substances (TBARS).

## Data Availability

Data are available on ClinicalTrials.gov (accessed on 1 February 2020).

## Supplementary Materials

The following are available online at https://www.mdpi.com/2072-6643/13/2/544/s1, Table S1: Study population baseline DXA, Table S2: Hand grip strength and physical activity at baseline and final visit.
