# Supplementary material for: U.S. Montmorency Tart Cherry Juice Decreases Bone Resorption in Women Aged 65–80 Years
Source: Nutrients. 2021 Feb 7;13(2):544. doi: 10.3390/nu13020544 (PMC7914470; doi:10.3390/nu13020544)
Supplement: Supplementary file 1 [file nutrients-13-00544-s001.pdf]

**Supplementary Table 1. Study population baseline DXA**

| Measure | TC1X[1] (*n*=14) | TC2X[1] (*n*=13) | *p*-value |
|---|---|---|---|
| Lumbar spine | | | |
| BMA ($cm^2$) | 56.3 ± 1.6 | 59.0 ± 2.1 | 0.319 |
| BMC (g) | 54.1 ± 2.6 | 55.0 ± 2.7 | 0.796 |
| BMD ($g/cm^2$) | 1.0 ± 0.04 | 0.9 ± 0.02 | 0.492 |
| T score | -0.8 ± 0.4 | -1.1 ± 0.2 | 0.494 |
| Total hip | | | |
| BMA ($cm^2$) | 35.6 ± 0.9 | 35.8 ± 1.4 | 0.892 |
| BMC (g) | 29.1 ± 1.5 | 28.6 ± 1.9 | 0.821 |
| BMD ($g/cm^2$) | 0.8 ± 0.03 | 0.8 ± 0.02 | 0.516 |
| T score | -1.0 ± 0.3 | -1.2 ± 0.2 | 0.428 |
| Femur Neck | | | |
| BMA ($cm^2$) | 5.1 ± 0.07 | 5.2 ± 0.1 | 0.535 |
| BMC (g) | 3.5 ± 0.1 | 3.5 ± 0.1 | 0.931 |
| BMD ($g/cm^2$) | 0.7 ± 0.02 | 0.7 ± 0.02 | 0.4470 |
| T score | -1.6 ± 0.2 | -1.7 ± 0.2 | 0.776 |
| Trochanter | | | |
| BMA ($cm^2$) | 11.4 ± 0.3 | 10.6 ± 0.3 | 0.051 |
| BMC (g) | 6.8 ± 0.4 | 6.4 ± 0.2 | 0.313 |
| BMD ($g/cm^2$) | 0.6 ± 0.02 | 0.6 ± 0.01 | 0.081 |
| T score | -1.1 ± 0.2 | -1.0 ± 0.1 | 0.865 |
| Intertrochanter | | | |
| BMA ($cm^2$) | 19.0 ± 0.9 | 20.0 ± 1.2 | 0.513 |
| BMC (g) | 18.8 ± 1.2 | 18.7 ± 1.7 | 0.965 |
| BMD ($g/cm^2$) | 1.0 ± 0.04 | 0.9 ± 0.02 | 0.189 |
| T score | -0.7 ± 0.2 | -1.2 ± 0.1 | 0.183 |

Data are presented as mean ± standard error (SE).
[1]TC1X = 1 fl. oz. tart cherry juice concentrate consumed once per day; TC2X = 1 fl. oz. tart cherry juice concentrate consumed twice per day at least 8 hours apart.
Bone mineral area (BMA), bone mineral content (BMC), bone mineral density (BMD).

**Scheme 2.** Hand grip strength and physical activity at baseline and final visit.

| | TC1X[1] ($n$=14) | | | TC2X ($n$=13) | | | $p$-value[3] | |
|---|---|---|---|---|---|---|---|---|
| Parameter | Baseline | Final | $p$-value[2] | Baseline | Final | $p$-value[2] | Base-line | Final |
| Hand grip strength[4] | | | | | | | | |
| Dominant hand | 11.5 ± 0.6 | 11.5 ± 0.6 | 0.904 | 12.3 ± 0.6 | 12.3 ± 0.7 | 0.976 | 0.397 | 0.408 |
| Non-Dominant hand | 11.8 ± 0.7 | 12.4 ± 0.7 | 0.123 | 11.6 ± 0.6 | 11.9 ± 0.7 | 0.480 | 0.827 | 0.647 |
| Weekly EE (kcal/week) | | | | | | | | |
| Exercise (1 x$10^3$) | 1.4 ± 0.3 | 1.6 ± 0.3 | 0.216 | 1.8 ± 0.5 | 1.5 ± 0.4 | 0.518 | 0.492 | 0.777 |
| Total (1 x $10^3$) | 6.4 ± 0.6 | 5.7 ± 0.6 | 0.257 | 8.3 ± 1.6 | 6.2 ± 0.9 | 0.058 | 0.280 | 0.645 |
| Month index score (unit/month) | | | | | | | | |
| Vigorous activity | 27.1 ± 4.9 | 28.2 ± 4.9 | 0.841 | 26.2 ± 4.0 | 13.8 ± 4.4 | 0.010 | 0.878 | 0.039 |
| Leisurely walking | 14.5 ± 2.9 | 16.3 ± 3.6 | 0.507 | 21.2 ± 3.1 | 12.9 ± 2.7 | 0.0020 | 0.123 | 0.466 |
| Summary Index | 62.6 ± 6.7 | 64.3 ± 7.2 | 0.702 | 67.8 ± 7.4 | 47.5 ± 7.5 | 0.001 | 0.604 | 0.119 |

Data are presented as mean ± standard error (SE).

[1]TC1X = 1 fl. oz. tart cherry juice concentrate/d; TC2X = 2 fl. oz. tart cherry juice concentrate/d

[2]*P*-values represent comparison within groups between baseline and corresponding final values.

[3]*P*-values represent comparison between TC1X and TC2X at baseline and final values.

[4]Grip strength assessed using a Jamar. Hand dynamometer are reported as the best of three attempts in kg of pressure exerted for each hand. Results are normalized to baseline kg lean mass in the corresponding arm (i.e., kg pressure/kg lean mass.
